# Supplementary material for: Design and evaluation of probe-capture sequencing for clinical Neisseria gonorrhoeae specimens
Source: medRxiv. 2026 Oct 30:2026.10.27.26366443. Preprint. [Version 1] doi: 10.64898/2026.10.27.26366443 (PMC13734223; doi:10.64898/2026.10.27.26366443)
Supplement: Supplementary file 1 [file NIHPP2026.10.27.26366443v1-supplement-1.pdf]

medRxiv preprint doi: https://doi.org/10.64898/2026.10.27.26366443; this version posted October 30, 2026. The copyright holder for this preprint (which was not certified by peer review) is the author/funder, who has granted medRxiv a license to display the preprint in perpetuity.
It is made available under a CC-BY 4.0 International license .

SUPPLEMENTARY MATERIALS

Supplementary Figure 1. Influence of amino acid distance threshold choice on assembly clustering dynamics

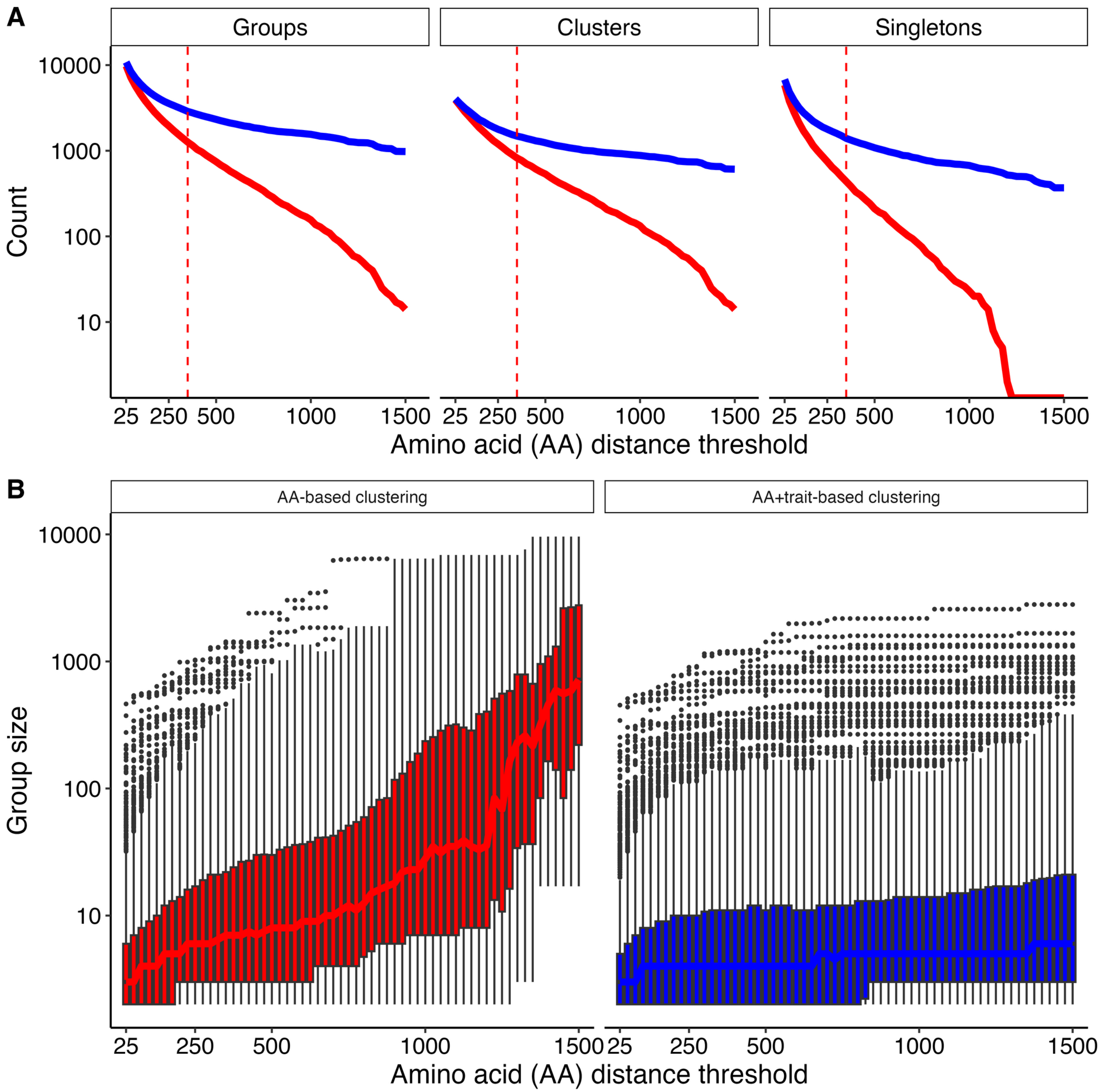

Genome assemblies were clustered at various core gene amino acid (AA) distance thresholds, ranging from 25 to 1,500. After partitioning assemblies into genetically related clusters (AA-based clustering), multi-assembly clusters were further partitioned based on their resistance-associated genotype and phenotype patterns (AA+trait-based clustering). (A) The number of groups across distance thresholds and the two genome clustering steps. Clusters were defined as groups with more than one isolate. (B) Distribution of group sizes across distance thresholds depicted as a box-and-whisker plot and faceted by clustering step.

medRxiv preprint doi: https://doi.org/10.64898/2026.10.27.26366443; this version posted October 30, 2026. The copyright holder for this preprint (which was not certified by peer review) is the author/funder, who has granted medRxiv a license to display the preprint in perpetuity.
It is made available under a CC-BY 4.0 International license .

Supplementary Figure 2. Overview of the Pathogenwatch *Neisseria gonorrhoeae* isolate collection

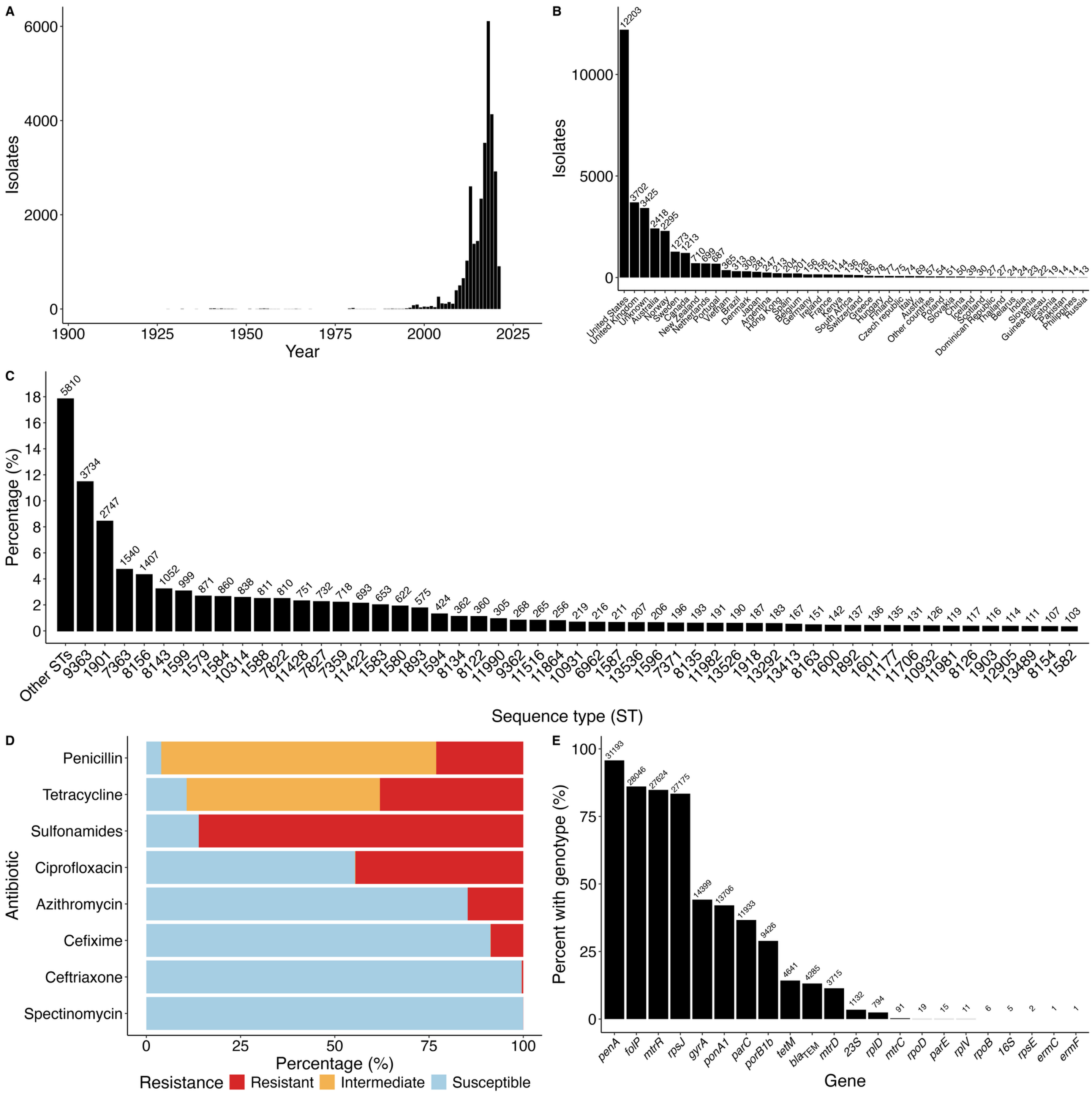

The global isolate collection comprises all publicly available whole-genome sequences available on Pathogenwatch at the time of analysis (downloaded on 11/24/2024). (A) Histogram of collection dates for selected genome assemblies. (B) Distribution of genome assemblies across countries. Countries with under ten assemblies were classified as ‘Other countries’ to aid visualization. (C) Proportion of assemblies by sequence type (STs). To aid in visualization, only the top 51 STs are represented. (D) Antibiotic susceptibility profiles of selected assemblies, categorized as resistant, intermediate, or susceptible, as predicted by Pathogenwatch. (E) Proportion of assemblies with resistance-associated genotypes, collapsed at the locus level.

medRxiv preprint doi: https://doi.org/10.64898/2026.10.27.26366443; this version posted October 30, 2026. The copyright holder for this preprint (which was not certified by peer review) is the author/funder, who has granted medRxiv a license to display the preprint in perpetuity.
It is made available under a CC-BY 4.0 International license .

568 **Supplementary Figure 3.** Phylogeny of the representative assemblies chosen for probe set generation

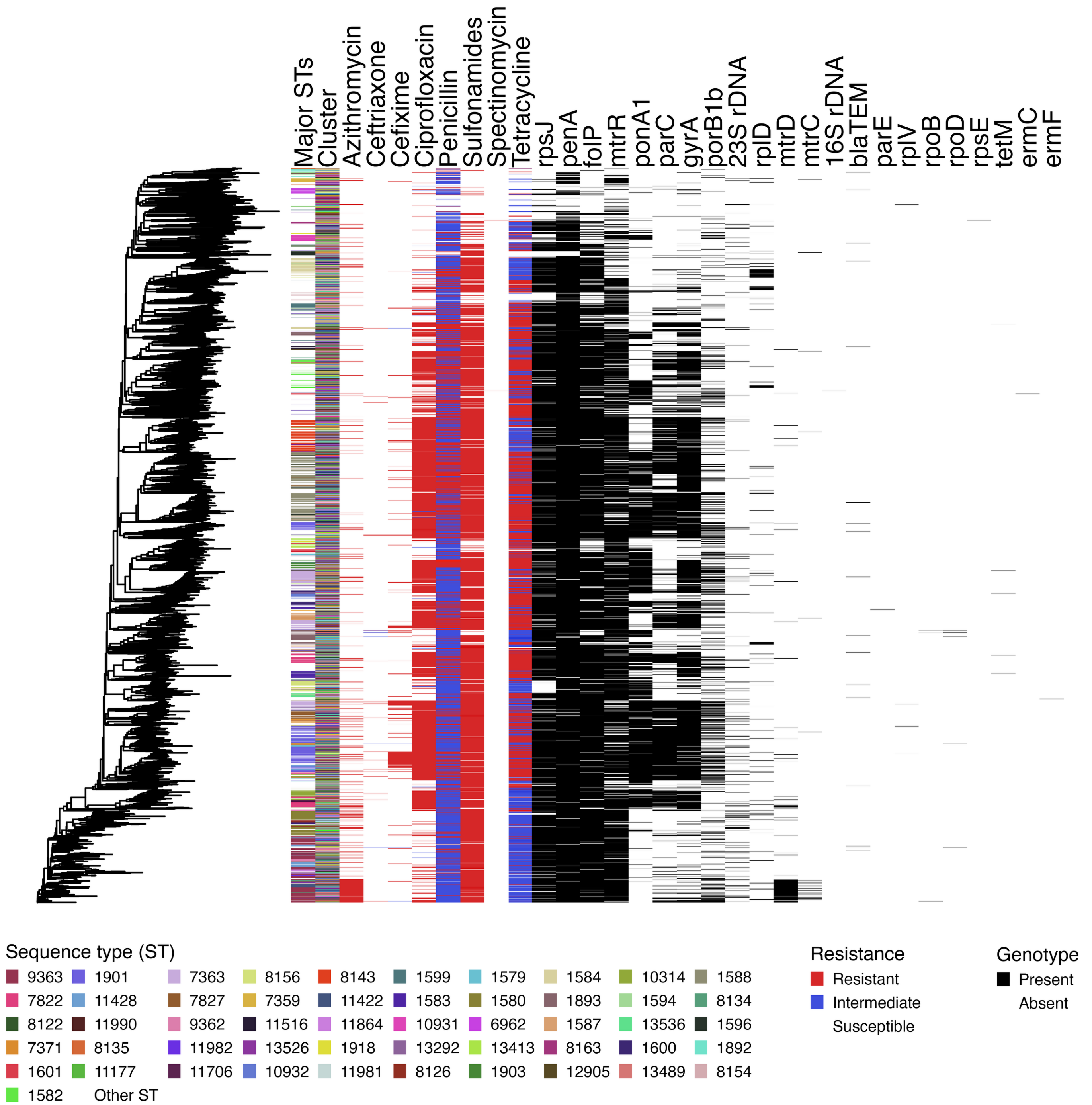

569

570 Phylogenetic visualization of the Pathogenwatch *Neisseria gonorrhoeae* assemblies used for probe set
571 generation. First, the top 51 sequence types (STs), determined by their frequency in the overall collection, were
572 displayed. Next, the amino acid distance clusters, inferred using a threshold distance of 350, were overlaid on
573 the phylogeny. Next, antibiotic susceptibility profiles were overlaid for eight antibiotics, classified as
574 susceptible, intermediate, or resistant, as predicted by Pathogenwatch. Finally, the presence of resistance-
575 associated genotypes, aggregated at the locus level, was overlaid on the phylogeny.

medRxiv preprint doi: https://doi.org/10.64898/2026.10.27.26366443; this version posted October 30, 2026. The copyright holder for this preprint (which was not certified by peer review) is the author/funder, who has granted medRxiv a license to display the preprint in perpetuity.
It is made available under a CC-BY 4.0 International license .

Supplementary Figure 4. Amino acid– and trait-based partitioning of *Neisseria gonorrhoeae* genomes

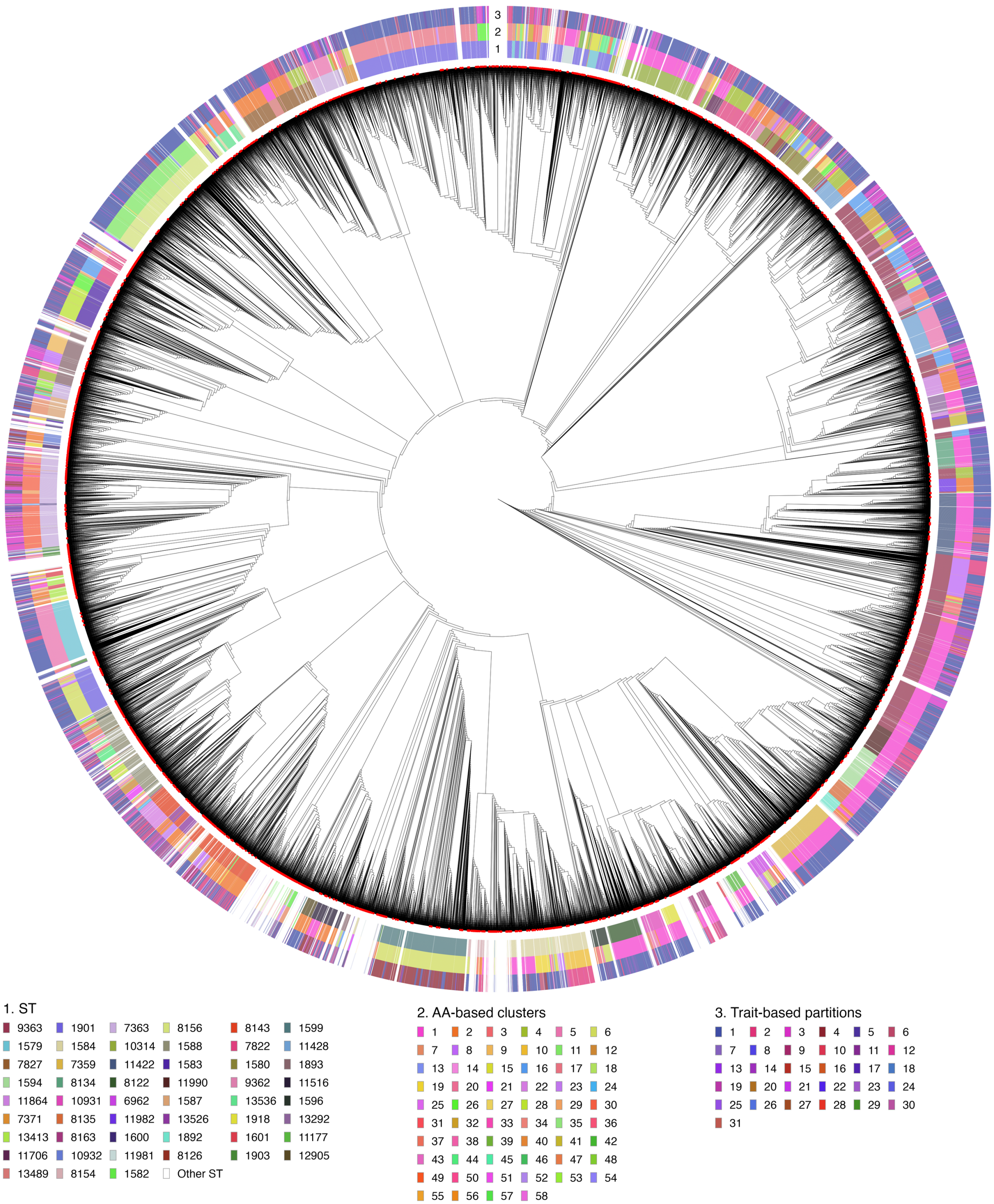

Phylogeny of publicly available whole-genome sequences available on Pathogenwatch at the time of analysis (downloaded on 11/24/2024). Amino acid distance-based clustering and subsequent partitioning of clusters by resistance-associated genotypes and phenotype predictions were performed to group clusters into representative groups. To facilitate representation of the groups, amino acid distance- and trait-based partitions are sequentially numbered within each sequence type. To simplify data visualization, partitioning data from the top 51 most abundant sequence types are provided.

medRxiv preprint doi: https://doi.org/10.64898/2026.10.27.26366443; this version posted October 30, 2026. The copyright holder for this preprint (which was not certified by peer review) is the author/funder, who has granted medRxiv a license to display the preprint in perpetuity.
It is made available under a CC-BY 4.0 International license .

Supplementary Figure 5. Overview of the *Neisseria gonorrhoeae* genomes selected for probe set generation

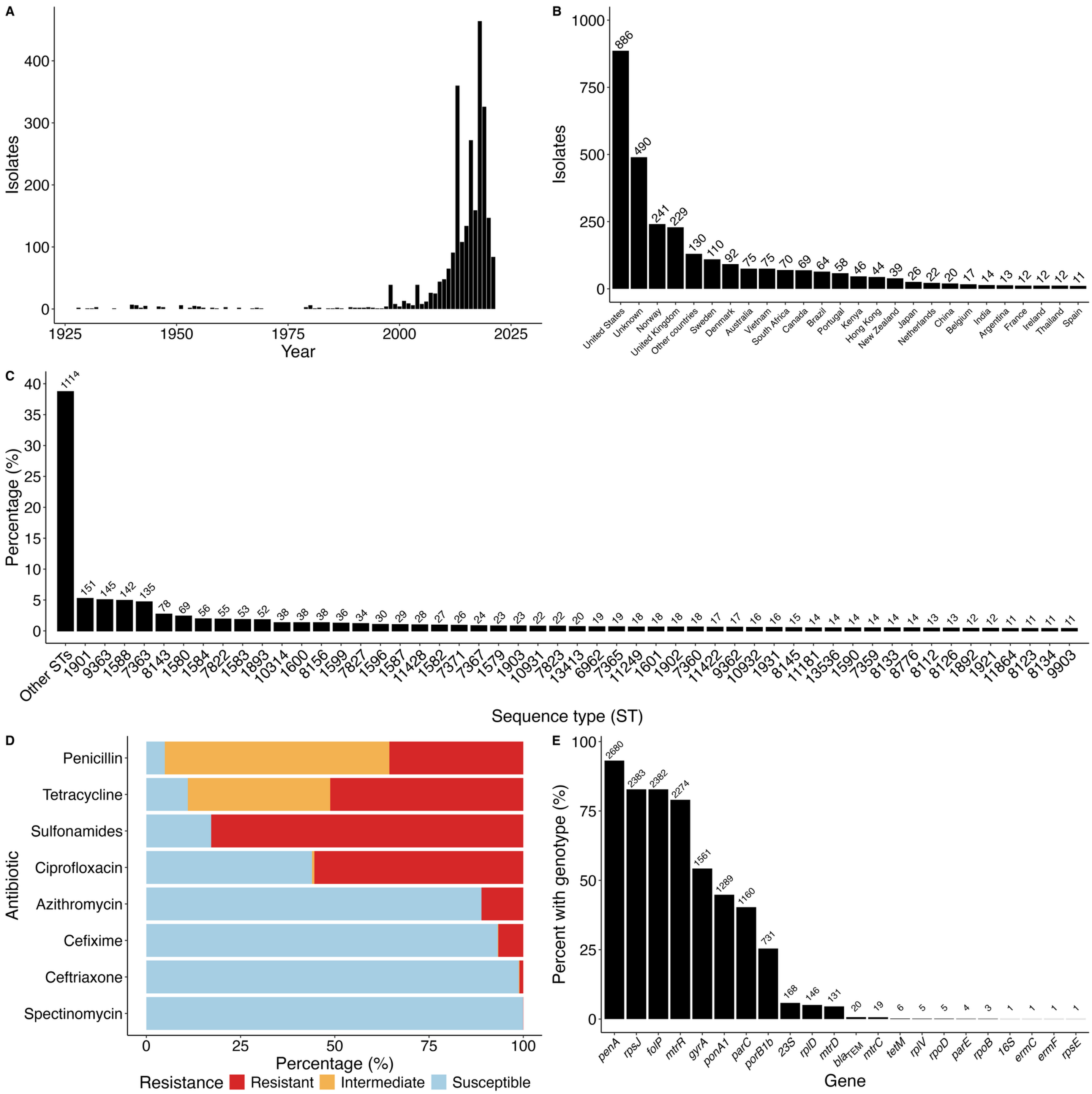

Representative isolates from Pathogenwatch, downloaded on 11/24/2024, were chosen using amino acid-based clustering and subsequent trait-based partitioning of cluster isolates. (A) Histogram of collection dates for selected genome assemblies. (B) Distribution of genome assemblies across countries. Countries with under ten assemblies were classified as 'Other countries' to aid visualization. (C) Proportion of assemblies by sequence type (STs). To aid in visualization, only the top 51 STs are represented. (D) Antibiotic susceptibility profiles of selected assemblies, categorized as resistant, intermediate, or susceptible, as inferred by Pathogenwatch. (E) Proportion of assemblies with resistance-associated genotypes, collapsed at the locus level.

medRxiv preprint doi: https://doi.org/10.64898/2026.10.27.26366443; this version posted October 30, 2026. The copyright holder for this preprint (which was not certified by peer review) is the author/funder, who has granted medRxiv a license to display the preprint in perpetuity.
It is made available under a CC-BY 4.0 International license .

Supplementary Figure 6. Representative genomes compared to the full *Neisseria gonorrhoeae* collection

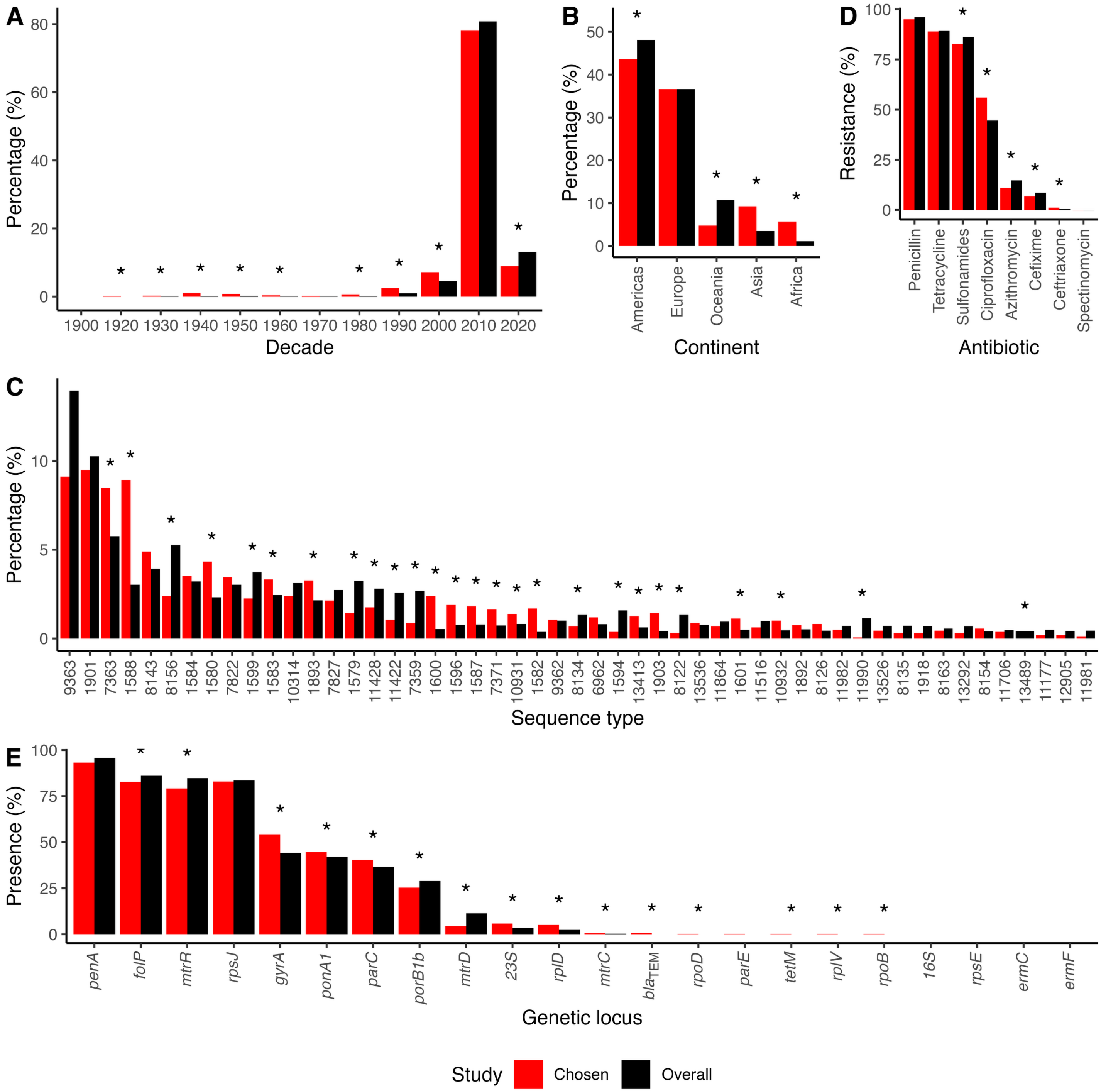

To evaluate the impact of partitioning assemblies using genetic distance and resistance-associated patterns, we compared the representative collection used for probe design to the overall Pathogenwatch collection: (A) temporal distribution of isolates by decade, (B) geographic distribution by continent, (C) sequence type (ST) distributions, (D) antibiotic susceptibility profiles, and (E) the distribution of resistance-associated loci at the gene level. Resistance was defined as a phenotypic prediction in the intermediate or resistant category. Asterisks indicate statistically significant differences between the chosen and global collection (Fisher's exact test, p-value <0.05).

medRxiv preprint doi: https://doi.org/10.64898/2026.10.27.26366443; this version posted October 30, 2026. The copyright holder for this preprint (which was not certified by peer review) is the author/funder, who has granted medRxiv a license to display the preprint in perpetuity.
It is made available under a CC-BY 4.0 International license .

Supplementary Figure 7. Coverage across resistance-associated reference loci at various read depth thresholds

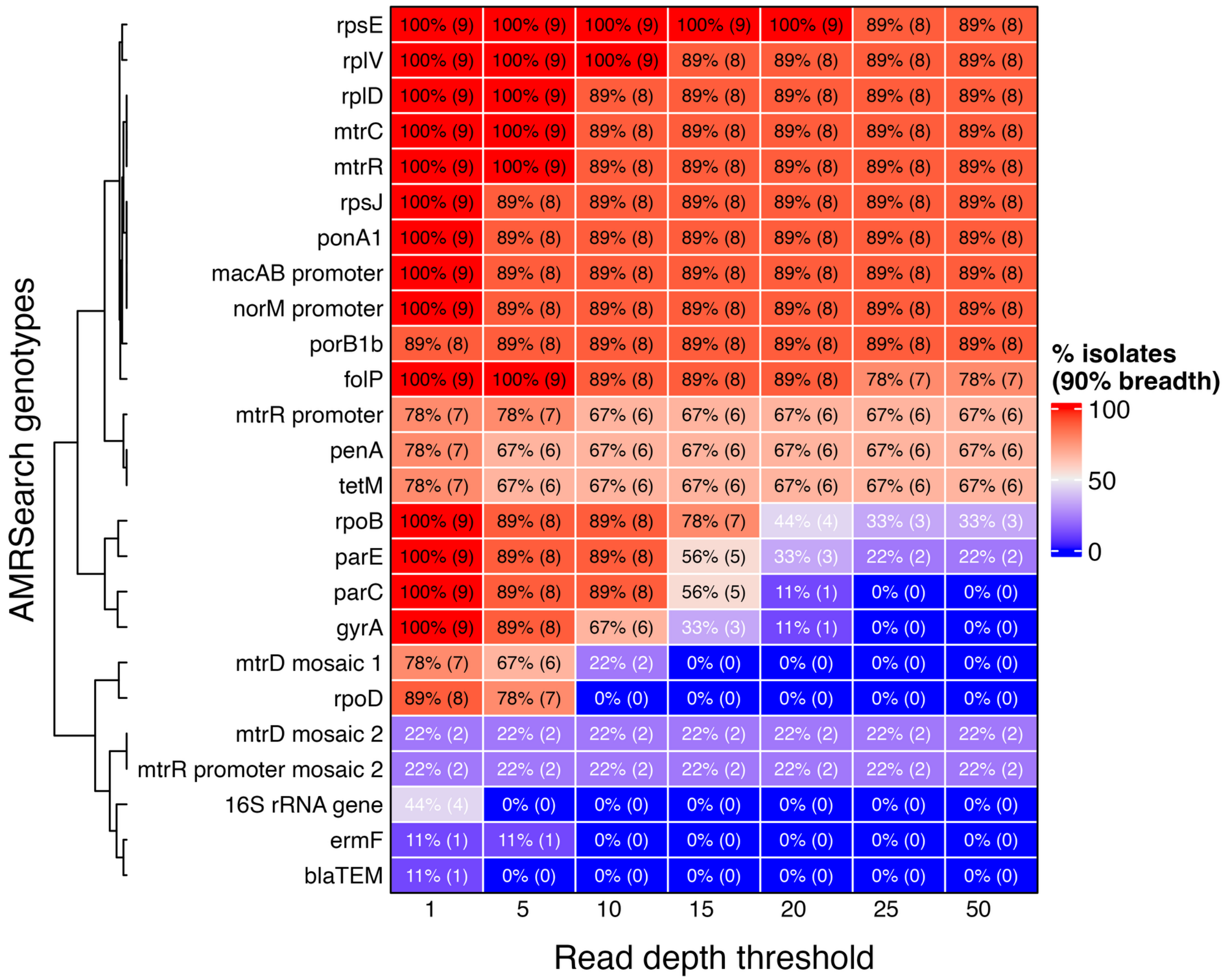

Read mapping was performed to evaluate coverage of Pathogenwatch's AMRsearch resistance-associated loci among nine *Neisseria gonorrhoeae*-positive urine cultures with high-quality assemblies. For each locus with at least one genome with non-zero coverage, the proportion of bases with read depth at or above the specified threshold was calculated. A locus was considered adequately covered if at least 90% met the depth threshold.

medRxiv preprint doi: https://doi.org/10.64898/2026.10.27.26366443; this version posted October 30, 2026. The copyright holder for this preprint (which was not certified by peer review) is the author/funder, who has granted medRxiv a license to display the preprint in perpetuity.
It is made available under a CC-BY 4.0 International license .

Supplementary Figure 8. Chromosome-normalized coverage across resistance-associated reference loci

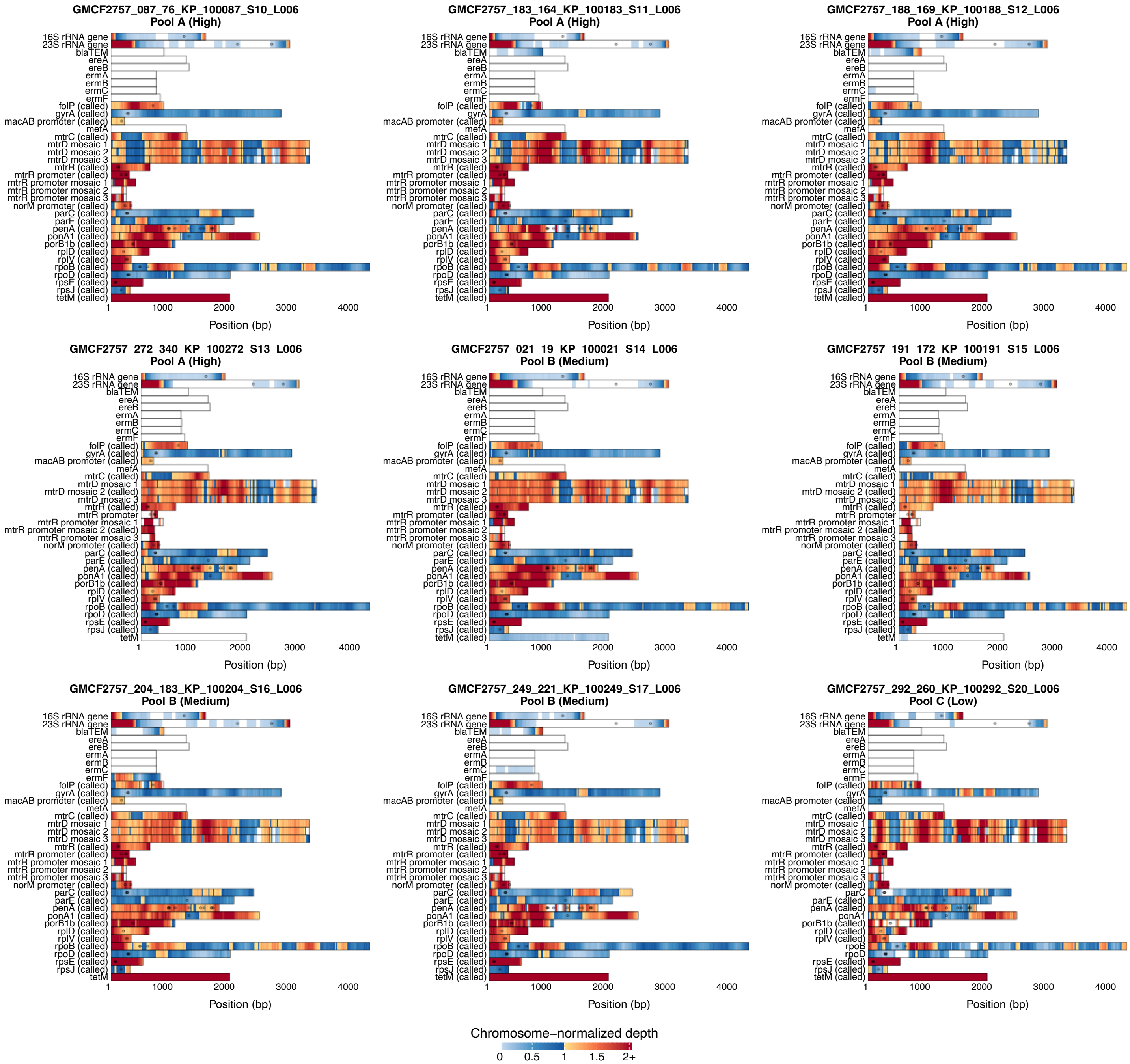

Read mapping was performed to evaluate coverage patterns of Pathogenwatch resistance-associated reference loci across nine *Neisseria gonorrhoeae*-positive urine cultures with high-quality genome assemblies. Each panel represents one isolate, with read depth at each position normalized using the median non-zero coverage when mapping reads to the well-characterized *N. gonorrhoeae* FA 1090 reference genome. Loci detected by AMRSearch are labeled "(called)", while circles denote mutation positions in the Pathogenwatch database, regardless of mutation detection.
